# An introduction to the special section on the Capability Approach to career guidance

**DOI:** 10.1007/s10775-021-09462-7

**Published:** 2021-02-20

**Authors:** Peter J. Robertson, France Picard

**Affiliations:** 1 Edinburgh Napier University, Edinburgh, Scotland; 2 Université Laval, Québec, Canada

**Keywords:** Career guidance, Capability approach, Social justice

## Abstract

This editorial introduces readers to the Capability Approach to career guidance. It outlines the origins of the approach in the work of economist Amartya Sen, and explains some of its key concepts. The Capability Approach offers a way to think about freedom, justice and well-being that has great relevance to the concerns of career guidance. A brief summary is provided of scholarship adapting the Capability Approach for application to career research, policy and practice. Finally, the four papers in the special section (Egdell & Robertson; Joncas & Pilote; Skovhus; Fuertes et al.) are introduced.

## Résumé

**Éditorial: Introduction à la section spéciale sur l'approche par les capabilités appliquée au développement de carrière** L’approche par les capabilités, qui a fait une percée dans le domaine du développement de carrière, tire son origine des travaux de l’économiste Amartya Sen. Elle renvoie à une idée de justice, ainsi qu’à une façon de concevoir la liberté et le bien-être des individus qui sont au cœur des enjeux en développement de carrière. Avant de présenter les contributions des auteures et auteurs, il importe de définir les concepts-clés de l’approche par les capabilités, ainsi que d’en illustrer l’utilisation tant en recherche, en intervention que dans les politiques liées au développement de carrière, à l’aide de quelques exemples. Quatre articles ont été retenus dans cette section spéciale, à savoir : Egdell & Robertson; Joncas & Pilote; Skovhus; Fuertes et al.

## Abstract (de)

**Eine Einführung zum Sonderteil des Capability Approach in der Berufsberatung** Dieses Editorial stellt den Lesern den Capability Approach in der Berufsberatung vor. Es umreißt die Ursprünge des Ansatzes in der Arbeit des Ökonomen Amartya Sen und erklärt einige seiner Schlüsselkonzepte. Der Capability Approach bietet eine Möglichkeit, über Freiheit, Gerechtigkeit und Wohlbefinden nachzudenken, die für die Belange der Berufsberatung von großer Bedeutung ist. Es wird eine kurze Zusammenfassung der wissenschaftlichen Arbeiten gegeben, die den Capability Approach für die Anwendung in der Laufbahnforschung, -politik und -praxis adaptieren. Schließlich werden die vier Beiträge des Sonderteils (Egdell & Robertson; Joncas & Pilote; Skovhus; Fuertes et al.) vorgestellt.

## Resumen

**Introducción a la sección extraordinaria sobre el Enfoque de las Capacidades aplicado a la orientación para el desarrollo de la carrera** Esta editorial presenta a los lectores el Enfoque de las Capacidades aplicado a la orientación para el desarrollo de la carrera. Esboza los orígenes del enfoque a partir del trabajo de la economista Amartya Sen, y explica algunos de sus conceptos clave. El Enfoque de las Capacidades proporciona una forma de concebir la libertad, la justicia y el bienestar que tiene gran relevancia para las cuestiones relacionadas con orientación para el desarrollo de la carrera. Se aporta un breve resumen de los estudios que adaptan el Enfoque de las Capacidades para su aplicación a la investigación, las políticas y la práctica sobre el desarrollo de la carrera. Por último, se presentan los cuatro artículos de la sección extraordinaria (Egdell & Robertson; Joncas & Pilote; Skovhus; Fuertes et al.).

## Introduction

We are delighted to bring you a special section in the *International Journal of Educational and Vocational Guidance* devoted to the Capability Approach to Career Guidance. Several of our authors participated in a symposium on the Capability Approach at the Annual Conference of the International Association for Educational and Vocational Guidance (IAEVG) in Gothenberg, Sweden in 2018. This successful event led to the proposal for the special section.

The section brings together contributions from Canada, the United Kingdom, and Denmark. Although very different in focus, they share a common source of inspiration in the Capability Approach. They also share a sense of its potential to contribute to our understanding of careers and to the design of career guidance approaches.

This editorial will introduce readers to the Capability Approach, and explain its relevance and potential value to the field. It will then briefly introduce the four articles in the section.

## Where does the Capability Approach come from?

The Capability Approach has its origins in the work of Amartya Sen. Born in Bengal in 1933, Sen is a Nobel prize winning Indian economist, whose academic work has primarily been in the UK and USA. His work can be located in the branch of the discipline that is known as ‘welfare economics’. Founded by British economist Arthur Pigou (1877–1959), welfare economics considers the overall benefit to society of all the economic decisions that people make. It is part of ‘normative economics’ where judgments (good or bad) are made about economic situations. It enables economic policies to be evaluated in terms of the impact they have on the well-being of the whole community.

One feature of Sen’s economics is a dissatisfaction with the use of crude financial measures, such as looking at an individual’s income as a way of judging how their life is going, or measuring Gross Domestic Product (GDP) to judge how well a country is doing. It is possible for a developing country to be undergoing rapid economic growth as measured by GDP, whilst wealth is shared unfairly, many people’s lives are unhappy, and social arrangements are unjust or oppressive. Sen explored alternative measures that give a more holistic and balanced view. He has been interested in what we mean by well-being, in addition to econometric measures. There has been growing interest in well-being as an important outcome of career guidance interventions (e.g. Redekopp & Huston, 2019; Robertson, 2013). As we will demonstrate, Sen’s conception of a good life has particular resonance with the concerns of career guidance.

Sen was influenced by personal experiences where he witnessed first-hand the impacts of famine in Bengal. He subsequently analysed the economics of the famine and found that it was inequity in food distribution, not food shortage that was root cause. There is a strong element to his work that seeks to address issues of injustice, albeit in a conceptually abstract way. Unusually for an economist, Sen’s work directly addresses philosophical questions, particularly in relation to what we mean by ‘freedom’ and ‘justice’ (e.g. Sen, 2009). Indeed, Sen might be considered to be a philosopher. Justice has been a concern throughout the history of career guidance field since its origins in the early 20th century, most obviously in the work of Frank Parsons (O’Brien, 2001). Notions of social justice are debated within current academic career discourses, and there are persuasive calls for practitioners and policymakers to give greater attention to equality issues (notably Hooley et al., 2017, 2018). Sen offers a distinctively different perspective on these debates by providing a conception of justice that places genuine freedom to make life choices at its core. This aligns well with the central concerns of career guidance.

The Capability Approach emerged from Sen’s work in the 1980s (Sen, 1980, 1985a, 1985b). Since he introduced these ideas, they have become widely influential and taken up by a variety of thinkers who have adapted them to a variety of applications including women’s rights (Nussbaum, 2000), international development work (Alkire, 2002), and education (Hart & Brando, 2018). As a result, the Capability Approach has grown and diversified since Sen’s original contributions.

## What is the Capability Approach? What are its key concepts?

As a starting point to considering social justice, Sen asked the question ‘*equality of what?*’ When societies grant rights and entitlements to citizens this does not necessarily lead to people enjoying the benefits that might flow from these privileges. For example, a level playing field in terms of legal rights to equality of opportunity in the labour market is available in many countries, but this does not necessarily mean that people can use these rights to their advantage, or enjoy equal outcomes in their careers. Genuine justice in practice is the goal for Sen; rights and entitlements may be necessary, but they are not sufficient.

A key distinction Sen identified was between having something, and being able to access the benefits it may be intended to confer. Possessing a resource (or a right) does not necessarily translate into being able to enjoy that resource as part of one’s lifestyle. A common example used to illustrate this distinction is that of a bicycle. Owning a bicycle is to possess a resource, but owning it is not adequate to enjoy the lifestyle benefits that this form of transport might confer. Additional factors are required to convert the resource into benefits, and these might include the skills to ride a bicycle, and an infrastructure of safe roads to ride on. Thus, Sen introduces a conceptual distinction between *resources* and *conversion factors*.

**Figure 1:**
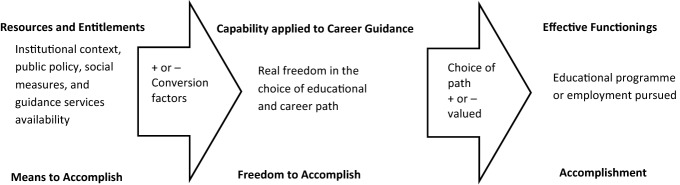
The usefulness of Capability Approach to career guidance. Adapted from Picard, Pilote et al. (2015)

Another key distinction is between *capabilities* and *functionings*. Individuals have certain resources available to that they can convert into lifestyles. A *capability set* is the full range of possible lifestyles that an individual can realistically access with the resources available to them: it defines the possibility space for their careers. *Functionings* relates to the actual current lifestyle they have achieved—it is the one potential reality of the capability set that has been implemented. Figure 1 shows how the conceptual components can be assembled to illustrate its relevance to the career guidance field.

For Sen, freedom means people being able to lead lives that they have *reason to value.* Justice means a genuine pragmatic access to valued lifestyles.

## How has the Capability Approach been applied to the study of careers and career guidance? What are the promising lines of research and practice in the field?

There have been calls for the Capability Approach to become part of the conceptual landscape of career guidance (e.g. Robertson, 2015; Robertson & Egdell, 2018). Although scholars have complained about the demanding task in operationalizing the Capability Approach, there is also growing body of empirical studies which use it as a conceptual lens through which to explore issues in the career guidance field. The authors of this special section refer extensively to these scientific studies. However, researchers usually focus on some of the capability concepts, but not all of them. Three examples of studies are discussed below.

Bonvin contributed to WorkAble (Otto et al., 2015), a transnational European project which applied the Capability Approach to address the needs of young people not in employment, education or training (NEETs). Bonvin’s focus was NEETs in Switzerland. He underlined the lack of consideration of what young people really valued (lack of a *capability for voice*). He pointed out how social policies did not take into account their right to refuse employment measures that did not fit their aspirations, without incurring certain sanctions such as a loss of an unemployment income (lack of *capability to exit*).

Poulsen et al. (2018) addressed the “Danish paradox”, that is the social mobility weakness in Denmark, compared to other Nordic countries, despite the provision of free education, social benefits, and healthcare. They observed that the career guidance practice, educational policy, and the political system tend to narrow young people’s opportunities. The authors emphasized the *choice* concept of the Capability Approach. They sought to promote social justice through widening opportunities for students (by raising awareness of options considered, set aside or rejected outright) before the educational and vocational choices.

Finally, Picard et al. (2019) highlighted the usefulness of the concept of *conversion factors* for counsellors during the transition to higher education in Canada (see also the Joncas & Pilote paper in this issue). The conversion of resources and formal rights into real options can open up students’ opportunities and expand their freedom. For example, first-generation university students tend to overestimate the tuition fees and ignore the financial measures designed for them. Therefore, career counsellors who provide the “right” information on the cost of university tuition and available financial assistance can represent an effective conversion factor. Picard et al. (2019) propose a further use of Capability Approach in guidance practice based on enabling vulnerable persons to identify resources and formal rights, and to make good use of them in their educational and vocational pathways. This application of the Capability Approach integrates the social policies and public measures into the counselling practice.

Turning now to different matters, the ongoing COVID-19 pandemic underlines the relevance of the Capability Approach in the field of career guidance as a promising theoretical perspective to study the exacerbated inequities into the education system and the world of work. Previously, we posited the famine in Bengal as the formal event that stimulated Sen’s work on justice. Famine and the COVID-19 pandemic are two types of disasters that affect people differently. A month before the pandemic, the International Labour Organization (ILO, 2020a) revealed the ongoing inequities in world of work according to socio-economic factors (e.g. gender, race) and vulnerability factors (e.g. occupational status, career stage). UNESCO (2020) provided data about the access to education in Sub-Saharan Africa during the pandemic: almost 90% of young students have not had access to a computer, nor to and Internet connection at home. This lack of informational resource is a real obstacle to learning for the most disadvantaged pupils and students, enhancing the social fracture between rich and poor. In Western nations, a recent body of studies has revealed how COVID-19 exposed inequities in workplace related to gender, working status or employment sector, immigrant status, race, and young people (Blustein et al., 2020; Cloutier-Villeneuve, 2020; Kantamneni, 2020; Kramer & Kramer, 2020; ILO, 2020b). Temporary workers, women and young people working in the services sectors have been the most affected by unemployment during the pandemic. Essential or frontline workers (e.g. cashiers in grocery stores; orderlies in long-term care facilities for the elderly), where women, immigrants and Black and Minority Ethnic (BAME) groups are overrepresented, have sometimes put their health at risk performing tasks without protective equipment.

For instance, those inequities can be analysed by the lens of Capability Approach: (1) the *capability for voice* for the most vulnerable groups affected by the pandemic; (2) the real access to the economic measures implemented to help people facing the lockdown and to safeguarding lives (*conversion factors*); (3) and the *capability to exit* unsafe working conditions.

## What added value does the Capability Approach bring to the study of career development, and the policy and practice of career guidance?

Most concepts in career theory derive from psychology or sociology. It is refreshing to consider a conception that emerges from economics, a different discipline that brings a very different perspective on an individual’s relationship with their career. It locates justice and capabilities (awareness of the possibilities that a person has reason to value) as central to the practitioners’ reflective practice and the working alliance with their clients (Guichard, 2008). The Capability Approach has a multilevel perspective, taking into account the micro, meso and macro level involved in a human situation or a scientific question (e.g. Berthet, 2015, 2016; Bonvin & Orton, 2009; Chiappero-Martinetti et al., 2015; Picard, Pilote et al., 2015). This scope enables researchers and practitioners to add a consideration of institutional context, public policy, and social measures into their analysis or practice.

The Capability Approach has a normative dimension derived from normative economics. Its key concepts can serve as guidelines to reflect on how to address the inequality or the discrimination issues and what should be the objectives of public policy (Berthet, 2015). The Capability Approach provides a distinctive perspective from which to engage with this debate. This helps us to acknowledge and analyse inequalities, and to promote the practices that give people a genuine opportunity to choose a life they have reason to value, and to reflect on those choices (recognising adaptive preferences defined as valuable by social or organisational norms) (Bonvin & Farvaque, 2005).

## The papers in the special section

***Egdell and Robertson*** provide a critique of the Capability Approach in relation to career guidance. Arguing that contemporary career theory tends to be presented uncritically, they seek to provide some balance to the arguments in favour of the Capability Approach presented in this editorial. They identify three main types of critique that could be made. Firstly, conceptual critiques relating to debates about what we mean by freedom and justice. Secondly, critiques relating to the limitations and scope of the discipline of economics. Thirdly, critiques relating to the considerable difficulties in operationalising the Capability Approach. The central issue emerging from this analysis is the incompleteness of the Capability Approach, and the need to combine it with other career concepts.

***Joncas and Pilote*** demonstrate how the Capability Approach can be useful to understand career development issues facing specific populations from a social justice standpoint. They focus on indigenous women in the Canadian higher education system. This is a group facing multiple challenges in their career development. Crucially, they highlight the way in which career guidance practitioners and services can represent conversion factors, when understood through the lens of the Capability Approach. The influence of these services can be either positive or negative—either facilitating or hindering the conversion of resources into desirable career outcomes.

***Skovhus*** demonstrates how the Capability Approach can be used to understand challenges and inspire solutions for career guidance practice. Focusing on the Danish secondary school system, she looks at initiatives in the design, delivery and evaluation of career guidance provision. Application of the Capability Approach ensures a strong focus on social justice concerns. In this example, the capabilities of students were enhanced by a shift away from a short-term focus on immediate decision making towards an exploratory approach and longer-term outlook. This enhances freedom.

***Fuertes, McQuaid and Robertson*** address the ideology underpinning public policy and practice for welfare-to-work programmes. They identify two main approaches which they characterise as ‘Work First’ (rapid placement into available employment) and ‘Human Capital Development’ (embed work skills in job seekers). They analyse three non-overlapping academic literatures that might inform a consideration of ‘employability’ for unemployed people on a welfare to work programme: the labour market literature, the career development literature, and the Capability Approach literature. By combining these perspectives, they generate an alternative approach to welfare to work that positions itself in opposition to the work first and human capital development conceptions. They label this a ‘career-first’ approach. This is an approach that advocates a deep embedding of career guidance concepts into welfare-to-work programmes, and supporting participants to access the lives they have reason to value.

## What can we take away from these contributions?

Whilst Sen addresses abstract concepts of freedom and justice, his understanding of well-being defines a good life in terms of a life that we have reason to value. This has great resonance with the way in which a good career is understood—it is something that we value. Career guidance is about helping people to do what is important to them, to pursue lives of work and study according to their own values. This does not imply a simplistic understanding of values. They may be individual to us but also part of our culture. Our priorities may evolve over time. They may sometimes be in conflict and we have to think what trade-offs must be made.

From this perspective career guidance is a pragmatic activity intended to help people convert the resources available to them into the lifestyle that they have reason to value. It means that guidance exists to promote freedom.
